# A cross-cultural adaptation and validation of a scale to assess illness identity in adults living with a chronic illness in South Africa: a case of HIV

**DOI:** 10.1186/s12981-022-00464-1

**Published:** 2022-08-21

**Authors:** Neo Phyllis Sematlane, Lucia Knight, Caroline Masquillier, Edwin Wouters

**Affiliations:** 1 School of Public Health, Faculty of Community and Health Sciences, University of the Western Cape, 5 Robert Sobukwe Road, Private Bag X17, Bellville, 7535 South Africa; 2 Centre for Population, Family & Health, Faculty of Social Sciences, University of Antwerp, Sint-Jacobstraat 2, BE-2000 Antwerp, Belgium; 3 Present Address: Division of Social and Behavioural Sciences, School of Public Health & Family Medicine, Faculty of Health Sciences, University of Cape Town, Anzio Road, Observatory, 7925 Cape Town, South Africa

## Abstract

The chronic illness trajectory and its outcomes are well explained by the concept of illness identity; the extent to which ill individuals have integrated their diagnosed chronic illness into their identity or sense of self. The capacity to measure illness identity in people living with HIV (PLHIV) is still relatively unexplored. However, this is potentially useful to help us understand how outcomes for PLHIV could be improved and sustained. This paper aims to explore the cross-cultural adaptation of a Belgian developed Illness Identity Questionnaire (IIQ) and validate the instrument using a sample of South African adults living with HIV. We followed a phased scale adaptation and validation process which included an investigation of conceptual, item, semantic and operational equivalence and also examined the psychometric properties of the IIQ. The concept of illness identity with its four factors; engulfment, rejection, acceptance and enrichment in PLHIV, was found to be relevant within this context. Five items from the original IIQ were excluded from the adapted IIQ due to either semantic insufficiency and/or inadequate measurement equivalence. The mode of administration of the IIQ was changed to accommodate current study participants. The original four factor 25-item model did not fit current data, however, a better contextualized, four-factor, 20-item model was identified and found valid in the current setting. The results showed adequate statistical fit; χ^2^/d.f. = 1.516, RMSEA = 0.076, SRMR = 0.0893, and CFI = 0.909. Convergent and discriminant validity were also tenable. The cross-cultural adaptation and validation of the IIQ was successful, resulting in the availability of an instrument capable of measuring illness identity in PLHIV in a high HIV prevalence and resource-constrained setting. This therefore addresses the paucity of information and expands on knowledge about illness identity.

## Introduction

Ubiquitous availability of and access to highly active antiretroviral treatment (HAART) by people living with HIV (PLHIV) has rendered HIV to become a manageable chronic illness. As a result, PLHIV on long-term therapy can anticipate average lifespan comparable to that of the general population [11, 40]. Achievement of this ideal however, is predicated on the assumption that PLHIV on ART will achieve lifelong adherence rates of 95% or more to treatments [49, 56].

Moreover, for many chronic illnesses including HIV, clinical outcomes such as adherence to treatments have become a measure against which success or failure of their management is evaluated [15, 38]. Specifically for HIV, it has become conventional for PLHIV to be coached on the importance of adherence to ART and viral suppression prior to entry into treatment [50, 65]. While this approach and knowledge sharing are essential components in the biomedical management of chronic illnesses, they model ill individuals as passive recipients of care and somewhat discount and disregard the ill individual’s experience, psychosocial evolution and growth related to living with a chronic illness [27, 72]

The importance of the experience of illness from the ill individual’s perspective is often overlooked [5, 63], yet, it’s understanding could complement the biomedical approach, should be considered when planning intervention aimed at improving outcomes for chronically ill individuals and be made a priority [4, 35, 48, 63, 73]. A key thesis in the illness experience is the conception of illness identity, a construct encompassing both (1) the adaptation to living with chronic illness and (2) the extent to which the ill individual has integrated their diagnosed chronic illness, in this case HIV, into their identity or sense of self [10, 46, 47, 70, 73]. Essentially, Illness identity is a response shift mechanism; a cognitive process, that chronically ill individuals may go through, as far as their contexts allow, to accommodate their illness and it eventually influences their perceived quality of life [17, 39, 60]. Be that as it may, determinants of HRQoL for PLHIV have been elaborated on elsewhere [12] and the current study intentionally focuses on illness identity and does not necessarily expound on HRQoL for PLHIV.

Substantial evidence, from other chronic illnesses, demonstrates that successful illness integration into identity may be correlated with positive patient outcomes such as psychological and physical functioning, fewer chronic illness-related problems, better treatment adherence, good health related quality of life and improved self-management of illness [36, 39, 46, 47]. Non-integration of chronic illness into the ill individual’s identity may conversely contribute towards poor overall self-management and sub-optimal adherence to treatments [1, 67].

In forwarding this notion, several authors have cited the potential of empowering and engaging patients and increasing their illness competence as potential strategies to improve clinical outcomes [5, 24, 73]. This is particularly relevant for the case of HIV and optimal adherence to treatments for PLHIV, especially in high prevalence, low resource contexts such as South Africa [73]. Common among chronically individuals who have acquired illness competence, are empowered and engaged, is that they have undergone a unique transformational process and have managed to merge the past non-ill self with the present chronically ill self and have created new identity that is positively adapted to living with the chronic illness. As a result, they are knowledgeable about and become accountable for their illnesses and its treatments and ultimately live well with the illness [5, 73].

While some literature exploring illness identity in HIV exists, this is focused primarily on the importance of identity transition and reformation work required by PLHIV [28, 37, 62, 68] and describes the process of integrating the illness into identity [7, 64]. Although useful, most of these studies are qualitative and explore only some components of illness identity [25, 68]. It is also notable that most are conducted within the global north [18, 37, 62, 68], with very few in high HIV-prevalence, resource-constrained contexts, where the application of the concepts could be most appropriate [13, 74].

Quantitative measurement of the illness identity construct has, until recently, proven complex. To address the gap, Oris et al. [47] created the Illness Identity Questionnaire (IIQ) that they argue measures illness identity in its entirety. The IIQ incorporates different psychological, sociological and health perspectives on illness and self-related variables. The IIQ encapsulates the construct of illness identity in four subscales: engulfment, rejection, acceptance and enrichment [46, 47]. Other measures exists, for instance, the illness self-concept scale (ISCS) to evaluate how the experience of living with HIV impacts self-concept included measures of self-growth and self-loss, which are similar to the enrichment and engulfment states respectively [22] but does not measure the acceptance and rejection aspects. The HIV meaningfulness scale (HIVMS) uses four items which could be likened to enrichment within the illness identity construct [3] and excludes three attributes; acceptance, rejection and engulfment. Whilst the scales may be reliable and expand on knowledge of aspects of illness identity, we selected the IIQ as it encapsulates all aspects of illness identity simultaneously.

Be that as it may, despite the progress in the measurement of illness identity made by the IIQ, there is limited application of this construct or any others to HIV, specifically in high HIV prevalence contexts like South Africa and there has been no articulation of cross-cultural adaptation and validation process of the IIQ. In addition, the IIQ was originally developed, validated and used in high income geographic settings such as Belgium [46, 47] and illness contexts such as diabetes, refractory epilepsy, congenital heart disease and with multisystem connective tissue disorders [39, 46, 47]. This then warrants adaption to the South African context [20, 26, 52].

## Study aims

The intention of the current study was therefore to achieve two objectives: (1) explore the cross-cultural adaptation of the IIQ and (2) present evidence for the concept of illness identity in PLHIV by evaluating the validity of the IIQ using a South African sample. To achieve this, we evaluated the factorial validity and reliability of the IIQ, with the aim to validate subscale scores on the IIQ in PLHIV, including assessing internal consistencies for the four illness identity subscales. We also assessed differences in illness identity based on age, gender and illness duration as was the case in the original validation of the instrument. To demonstrate external validity of the instrument, we explored the association of the different illness identity subscales with HIV related stigma and disclosure of HIV status.

## Methods

### Data source, collection and participants

The current validation study forms part of a larger research study which aims to investigate illness identity in PLHIV, entitled *HIV Illness Identity, Household HIV Competence and Antiretroviral Treatment Adherence: an analysis of associations among treatment naïve adult population living with HIV* and for which a detailed research proposal has been articulated. The illness identity in HIV research study is in turn embedded within the Sinako cluster randomised control trial [41, 42]. The Sinako trial and intervention are described elsewhere [41, 41, 42, 42]. The trial aims to use community healthcare workers (CHWs) to stimulate HIV competence among PLHIV and the households, and explore the impact of the intervention on ART adherence outcomes and in turn enhance the routine support provided by CHWs [41, 42].

Data was obtained from the baseline phase of the trial. Due to the global COVID-19 pandemic and subsequent national lockdowns that were instituted by governments however, data collection activities had to be suspended until it was deemed relatively safe to continue. The study setting is five sub-districts of the Cape Metro health district in the Western Cape, South Africa; Mitchell’s Plain, Khayelitsha, Klipfontein, Eastern and Western. Unemployment is widespread and these communities are largely poor [55]. HIV prevalence in the Cape Town metro was estimated at 21.6% in 2015. Khayelitsha sub-district has been identified to have the highest overall HIV prevalence rate, 29.5% and 34.3% among pregnant women within the Western Cape province [33, 61]

Following a process of adaptation and development of a baseline questionnaire, data was collected from adults living with HIV and relatively recently started on ART visiting the 12 participating healthcare facilities and were recruited for participation in the study. Baseline data was collected between 8th October 2019 and 13th March 2020 by trained fieldworkers from 152 PLHIV who all provided informed consent. Data collection activities were then suspended due to the global COVID-19 pandemic which resulted in a total national lockdown. Ethical clearance to conduct the study was obtained from both the University of the Western Cape’s Biomedical Research Ethics Committee (BM19/4/6) and the Ethical Committee for the Social Sciences and Humanities of the University of Antwerp (SHW_17_64). Permissions to access health facilities were also issued by the Western Cape Department of Health and the City of Cape Town [41, 42].

### Process for adaptation and validation

This study employs an approach initially proposed by Herdman et al. [26] as a systematic guide to direct the cross-cultural adaptation and validation process for the IIQ (Table 10 in Appendix) [20, 52]. With this approach, the constructs: illness identity, and its sub-scales acceptance, rejection, enrichment and engulfment, are assumed to be different across contexts, in the current case, Belgium and South Africa. It is therefore necessary to explore whether the concepts exist and whether they are interpreted the same way in this context as was in the original context [26, 52]. This process involved four-phases.

The first phase employed a literature review to evaluate conceptual and item equivalence of the illness identity construct and its domains in the target context. For this, we undertook a general literature review using the EBSCOhost interface, which provided access to a number of databases and the PubMed database. We followed a process of keyword, backward and finally forward searching to retrieve relevant published articles. This process gathered information from studies that were of relevance, important and valid for the illness identity construct. Using critical analysis, we especially scrutinised the literature for papers on illness identity, its related or similar concepts and or its four domains of acceptance, rejection, denial and enrichment, published in both target (South African or similar) and original contexts. The approach not only provided basis and the rationale for the overall illness identity study, but in this way, we could evaluate whether the illness identity domains had the same relationship and meant the same thing in both the original and target context. Additionally, we conducted a workshop with research fieldworkers, who represented the target population for our research study, to explore meanings of items within the IIQ from an emic perspective and advise on their appropriateness, relevance and acceptability [26, 52].

The second phase explored semantic equivalence using the technique of adaptation, also referred to as cultural substitution [43], to translate the instrument from the original English into the relevant local languages; *isiXhosa* and Afrikaans. This involved replacing the original text with appropriate local language making the text easy to comprehend. In some cases, the borrowing translation technique was used [43]. This is when direct translation with a word or phrase made the translation unnecessarily formal or complex and local speakers are likely to understand the original language (in our case, English) word. The technique was used only in a few instances where it was impossible to substitute a word. The translation process involved both forward and back-translations. The initial translation of the IIQ into *isiXhosa* was conducted by a first language *isiXhosa* speaker who is also fluent in English. This version was reviewed by another person fluent in both languages. Thereafter, two independent translators back translated the *isiXhosa* version to English. The two back translated versions were then compared against the original IIQ using two measures; comparability of language and similarity of interpretability by a person fluent in English [59]. For this, a seven-item Likert scale ranging extremely comparable/similar), through moderately comparable/similar to not at all comparable/similar was used.

The third phase assessed the operational equivalence and explored the potential for using the original questionnaire format, instructions, mode of administration and measurement methods in the context.

In the fourth and final phase, we assessed validity by exploring the psychometric properties of the IIQ. This involved three steps: (1) evaluation of the factorial validity and reliability; (2) given that there were no differences in illness identity based on age, gender and illness duration in the original validation of the IIQ using adolescents and emerging adults with type 1 diabetes [47], we also aimed to investigate whether consistency of the results for these two demographic variables and one clinical parameter could be established in adults living with HIV, and (3) finally, we explored association of the different illness identity subscales with HIV related stigma and disclosure of HIV status. We hypothesized that acceptance and enrichment would be positively correlated and that rejection and engulfment be negatively associated with disclosure of HIV status and HIV related stigma [7, 73].

### Measures

#### Illness identity questionnaire

The IIQ has four illness identity subscales: engulfment, rejection, acceptance and enrichment, represented by 25 items and participant responses are captured on a five-point Likert scale ranging from “strongly disagree” to “strongly agree” [46, 47].

When engulfed by illness, chronically ill individuals describe themselves according to their illness, they perceive the illness to be intruding in all areas of their lives and the illness seems to control their identities and routines, compromising other significant self-assets [46, 47]. Rejection on the other hand describes the extent to which the illness is rejected as part of their identity [46, 47, 70]. As such, the rejection and engulfment scales capture the lack of integration into self of the chronic illness. The engulfment subscale consists of eight items and the rejection subscale five items [46, 47].

Acceptance is the degree to which individuals accept their illness as a part of their identity, regardless of other social roles and identity assets. With acceptance, chronically ill individuals acknowledge their illness, they are not overwhelmed by it and the illness does not pervade other areas of their lives. In fact, with the acceptance process, they try to live their lives as normal as their illnesses allow and the illness plays an insignificant role in their lives [39, 44, 46, 47]. Enrichment indicates the extent to which having a chronic illness has either resulted in positive life changes, benefited the ill individual’s identity, and or facilitated their personal growth [46, 47, 70]. To this end, the acceptance and enrichment states represent more adaptive illness integration. The acceptance subscale consists of five items and the enrichment subscale is comprised of seven items [46, 47, 70]. The IIQ has been validated and used previously to measure illness identity in high income contexts among individuals with chronic illnesses including diabetes, refractory epilepsy and congenital heart disease [39, 47, 69, 70].

#### HIV disclosure questions

Two aspects of disclosure were explored: participants’ inclination to disclose their HIV status and situational disclosure. Inclination to disclose was estimated by how strongly participants agreed with the statements that assess the comfort and perceived ability to reveal their HIV status and situational disclosure by participants indicating how strongly they agreed or disagreed with a statement about comfort but only when necessary.

#### HIV related stigma

For HIV related stigma, we used the truncated HIV stigma scale previously validated by Reinius et al. [53], adapted from a longer 40-item HIV stigma scale [9]. Whilst other stigma scales such as the “People Living with HIV Stigma Index” [19] exist, we found the utility of the shortened scale appropriate for our context since the focus of our inquiry was to determine external validity of the IIQ and not stigma per se. The 12-item version is comprised of four subscales; personalised stigma, disclosure concerns, concerns about public attitudes and negative self-image measured by three items each [53].

### Statistical analysis

The IIQ has been previously validated [47] and therefore as an initial step, we conduct a confirmatory factor analysis (CFA). The condition is that if the original model fits the data then it will be accepted and adopted as is with the item and or semantic and operational adjustments that are made for the study context described above. To evaluate model fit, we use a 2-index presentation strategy and because our sample size was relatively small (N ≤ 250), we adopt the combinational rule based on the comparative fit index (CFI) > 0.96 in combination with the Standardized Root Mean Squared Residual (SRMR) < 0.09 [30]. For transparency, we also evaluate and compare other fit indices obtained in the current study against those that were used and assessed in the original IIQ validation. These include the normed chi square, which is the ratio of the chi-square statistic to the respective degrees of freedom (χ^2^/DF), the cut-off is set at less than 2 and the root mean square error of approximation (RMSEA), a parsimony adjusted index, which should be less than 0.08 [46].

As a contingency, we conduct an exploratory factor analysis (EFA) of the original 25-item model, evaluating it for any redundant items [32, 71]. For the EFA, to evaluate factorability of the data, sample adequacy and non-randomness of the correlation matrix criteria were used. For this, the Kaiser–Meyer–Olkin (KMO) statistic [34], which is required to be above 0.50 and the Bartlett’s test of sphericity [6], required to have a significant p-value, are used. The correlation matrix is then submitted for exploratory factor analysis. Because our intention was to examine the factor structure of the IIQ rather than a reduction exercise, we use the common factor analysis instead of the principal component analysis. For this we elect principal axis factoring extraction method with initial communalities estimated by squared multiple correlations which has been demonstrated to be relatively accommodative of non-normality of the data distribution and has capacity to retrieve weak factors [71]. As recommended we use the visual scree test and parallel analysis to determine the number of factors to retain [71]. Although a common practice, we elect not to use eigenvalues to determine the number of factors to retain as the method is cited as often inaccurate and its use is discouraged [71]. Furthermore, an oblimin rotation is employed because of the understanding that the factors are indeed correlated. Redundant items are defined as those items with: pattern coefficients < 0.5, or with cross/complex loadings that were salient (> 0.4) on more than one factor. In these cases, the item is removed from the model to fulfil the simple structure principle [66, 71]. For a factor to be considered adequate, it is to have a minimum of three theoretically meaningful and salient pattern coefficients > 0.4. We then recalculate the adjusted model and submit it for another CFA. We re-specify the model by progressively removing the items with poor standardised factor loadings and reassess its goodness of fit with the data. We eventually undertake an exploratory phase of inspecting the modification indices of all the pairs of error terms and correlating those pairs with the largest indices until the model fitted [2].

Reliability, convergent and discriminant validity of the final measurement model is assessed using different estimates including correlations, the average variance extracted (AVE) which should be ≥ 0.5 and composite reliability (CR) which should be ≥ 0.7. Although the CR is a less biased estimate of reliability than Cronbach’s Alpha, we also report on the latter estimate. Multivariate analyses of variance (MANOVA), using Wilks’ Lambda, are also used to test for mean differences in illness identity as dependent variable based on gender. For age and illness duration, Pearson correlation coefficients are calculated with the four illness identity states. To examine the associations linking illness identity to aspects of disclosure of HIV and internalised stigma, Kendall’s (τb) and Spearman’s correlation coefficients are calculated. All analyses were conducted with the IBM SPSS Statistics for Windows, (Version 27.0.; released 2020) and the IBM SPSS AMOS 27.0.0 package (Version 4.0.30319.42000).

## Results

### Adaptation of the IIQ to the South African context

#### Investigation of conceptual and item equivalence

A comprehensive literature review revealed that the four constructs within the IIQ were etic and could be discerned in the target context [16, 31]. Although studies that specifically used the IIQ questionnaire were not identified, a number of studies, mostly using qualitative methods and conducted in similar contexts as the current study, reported the IIQ constructs (such as acceptance, rejection or denial and positive self-concept) as emerging themes [23, 54, 57, 58, 74]. We note however, that expression of these constructs by individuals from different cultural contexts may be significantly emic. Furthermore, feedback by the fieldworkers who represented the target population and reviewed the instrument items for meaning and relevance, was that all the original IIQ items were appropriate for the context. Therefore, all items of the IIQ were retained in this phase and no other items were added to the instrument.

#### Evaluation of semantic equivalence

Following implementation of the translation process as described above, five items from the original IIQ were back translated inconsistently by both translators. For the two items “I refuse to see my HIV as part of myself” and “because of my HIV, I have learned a lot about myself”, the word “myself” was translated as “my body” by both back translators. “Simply belongs to me” from the item “My HIV simply belongs to me as a person” was also problematic. The back translators interpreted the phrase as “is mine simply” and is “is easily mine” respectively. The phrase “being talked to” from the item “I hate being talked to about my HIV” also proved to be fiddly for translation because it was back translated as “when I have to have my HIV discussed and “they talked to me about my HIV. The final item, “My HIV dominates my life” was back translated as “My HIV status causes my life to be what it is” by just one of the translators. Because comparability of language and or similarity of interpretability were compromised for these five items, the items had lost their original meaning. The meaning of the rest of the other twenty items was not lost. Because each stage of the adaptation and validation exercise was independent, all 25 items were move to the psychometric evaluation phase but flagged, despite this result.

#### Assessment of operational equivalence

We elected to retain the questionnaire format, its instructions and measurement methods as was in the original setting. The mode of administration however was amended. Trained isiXhosa first language research assistants administered the IIQ instead of it being self-administered. This was to accommodate the literacy level of the population. In addition, given that the IIQ was assessed along with other items and scales the average length of time it took to administer the questionnaire was not the same as it was in the original context.

### Validity of the IIQ

#### Sample

Of the 152 respondents who consented to participate in the study, we obtained a total sample of 90 IsiXhosa speaking respondents. There were no missing data. Of this sub-sample, 73% were females with a mean age of 30 years while males had a mean age of 36 years. Almost half of the participants had only secondary education and 46% were unemployed. The average illness duration, that is time since first diagnosis at the time of data collection, was just under 2 years (Table 1).

**Table 1:** Participants’ characteristics

	All	Female	Male
Gender (%)		73.33	26.67
Age (in years)	31.91 (9.80)	30.33 (8.98)	36.25 (10.82)
Illness duration (in months)	20.45 (43.63)	22.06 (42.54)	15.91 (47.26)
Education level (%)			
None	1.1	0	4.2
Primary	25.6	21.2	37.5
Secondary	43.3	47	33.3
Matric	28.9	30.3	25
Diploma/university	1.1	1.5	0
Employment status (%)			
Full time	15.6	7.6	37.5
Part time	33.3	6.1	16.7
Casual	6.7	12.1	8.3
Pensioner	1.1	0	4.2
Unemployed (studying)	10	12.1	4.2
Unemployed (and willing and able to work)	45.6	56.1	16.7
Unemployed (unable to work)	7.8	6.1	12.5

#### Initial confirmatory analysis of the original 25-item IIQ

The original four-factor, 25-item illness identity scale (original model) did not fit the current data; model chi-square test statistics = 532.147, degrees of freedom = 269, probability level ≤ 0.001, normed chi-square test = 1.978 and comparative fit index (CFI) = 0.761. RMSEA = 0.105 and SRMR = 1.065 (Table 2). In addition, two items “I never talk to others about my HIV” and “My HIV simply belongs to me as a person”, had standardised factor loadings < 0.4 (0.331 and 0.285 respectively), indicating that the two items were weakly correlated with their latent factors of engulfment and acceptance (Table 3). Of note, the item “My HIV simply belongs to me as a person”, is one of the five items that had lost meaning, as described above, at the semantic evaluation phase.

**Table 2:** Fit indices of the different tested models

Model	Chi square	Degrees of freedom (DF)	Probability (> 0.05)	Normed χ^2^ (χ^2^/DF) (< 2)	RMSEA (< 0.08)	SRMR (< 0.09)	CFI (> 0.9)
Fit of 25-Item (original model) to current data	532.147	269	< 0.01	1.978	0.105	0.107	0.761
Fit of EFA informed, 22-Item model (Model 1) to current data_3 items deleted	422.184	203	< 0.01	2.08	0.11	0.103	0.788
Fit of EFA Informed, 20-item model to current data (final model)_further 2 items deleted and modification indices applied	244.061	161	< 0.01	1.516	0.076	0.089	0.909
Fit of original model (25-Item) to *Belgian data*	382.82	266	≤ 0.01	1.44	0.046	0.067	.909

**Table 3:** Standardised factor loadings for alternative models of the IIQ

Item	Original Model (25-item)	Model 1 (22-item)	Model 2 (20-items)
Rejection			
I refuse to see my HIV as part of myself	0.401		
I never talk to others about my HIV	0.331		
I'd rather not think of my HIV	0.700	0.658	0.651
I hate being talked to about my HIV	0.525	0.482	0.476
I just avoid thinking about my HIV	0.801	0.895	0.906
Acceptance			
My HIV simply belongs to me as a person	0.285		
My HIV is part of who I am	0.448	0.441	
I accept being a person with HIV	0.872	0.876	0.841
I am able to place my HIV in my life	0.944	0.943	0.988
I have learned to accept the limitations imposed by my HIV	0.587	0.582	0.544
Engulfment			
My HIV dominates my life	0.536	0.534	0.496
My HIV has a strong impact on how I see myself	0.479	0.478	
I am preoccupied with my HIV	0.684	0.684	0.601
My HIV influences all my thoughts and feelings	0.696	0.695	0.609
My HIV completely consumes me	0.782	0.785	0.821
It seems as if everything I do, is influenced by my HIV	0.841	0.843	0.911
My HIV prevents me from doing what I would really like to do	0.741	0.739	0.737
My HIV limits me in many things that are important to me	0.709	0.707	0.628
Enrichment			
Because of my HIV, I have grown as a person	0.752	0.753	0.782
Because of my HIV, I know what I want out of life	0.813	0.815	0.833
Because of my HIV, I have become a stronger person	0.825	0.825	0.836
Because of my HIV, I realise what is really important in life	0.698	0.696	0.704
Because of my HIV, I have learned a lot about myself	0.677	0.676	0.607
Because of my HIV, I have learned to work through problems and not just give up	0.744	0.744	0.691
Because of my HIV, I have learned to enjoy the moment more	0.652	0.651	0.625

#### Omitted items following exploratory factor analysis

As a result, to reassess the model structure, the original 25-item model was submitted for an EFA which revealed a four-factor solution with three definitively redundant items (Table 4). Two of the items *I refuse to see my HIV as part of myself* and *My HIV simply belongs to me as a person* had loadings that were salient on more than one factor and had poor factor loadings below 0.4 (Table 4). The same two items had also lost meaning as previously detailed at the semantic evaluation stage. The third item *I never talk to others about my HIV* had a standardised factor loading below 0.4 (Table 4). Therefore, because of loss of meaning, cross and poor factor loading, the three items, two of which were part of the rejection subscale, were subsequently deleted.

**Table 4:** Descriptive statistics and pattern coefficients for 90 IsiXhosa speaking participants on the rejection, acceptance, engulfment and enrichment items of the IIQ

Item	Descriptive statistics				Factor				
	Mean	Std. deviation	Skewness	Kurtosis	Enrichment	Engulfment	Rejection	Acceptance	Communality
I refuse to see my HIV as part of myself	2.7	1.185	0.234	− 1.362	− 0.034	**0.284**	**0.327**	− 0.04	**0.35**
I'd rather not think of my HIV	3.29	1.052	− 0.667	− 0.963	0.082	0.011	**0.709**	− 0.1	0.609
I never talk to others about my HIV	3.74	0.955	− 1.047	0.683	0.074	− 0.045	**0.36**	− 0.036	**0.312**
I hate being talked to about my HIV	2.67	1.081	0.813	− 0.616	− 0.17	0.269	**0.4**	0.031	0.404
I just avoid thinking about my HIV	3.19	1.101	− 0.178	− 1.273	− 0.163	0.005	**0.794**	− 0.063	0.63
My HIV simply belongs to me as a person	3.9	0.75	− 1.303	2.086	0.157	− 0.152	**0.394**	**0.315**	0.517
My HIV is part of who I am	4.01	0.662	− 1.676	6.219	0.093	− 0.057	0.045	**0.473**	0.632
I accept being a person with HIV	4.23	0.582	− 0.772	3.331	− 0.038	− 0.016	− 0.176	**0.877**	0.788
I am able to place my HIV in my life	4.16	0.598	− 0.709	2.681	0.176	0.025	− 0.214	**0.785**	0.815
I have learned to accept the limitations imposed by my HIV	4.1	0.619	− 0.645	1.995	0.131	− 0.02	0.013	**0.588**	0.539
My HIV dominates my life	2.26	1.127	1.017	0.235	− 0.139	**0.622**	0.099	0.335	0.57
My HIV has a strong impact on how I see myself	2.84	1.121	0.461	− 1.087	0.209	**0.608**	− 0.037	0.161	0.643
I am preoccupied with my HIV	2.39	0.92	1.176	0.556	0.239	**0.723**	− 0.086	− 0.133	0.628
My HIV influences all my thoughts and feelings	2.52	1.019	0.622	− 0.885	0.11	**0.678**	0.069	− 0.265	0.675
My HIV completely consumes me	1.94	0.866	1.596	3.35	− 0.088	**0.774**	− 0.15	− 0.001	0.711
It seems as if everything I do, is influenced by my HIV	1.9	0.671	1.486	5.791	− 0.103	**0.781**	− 0.014	− 0.024	0.788
My HIV prevents me from doing what I would really like to do	2.07	0.776	1.506	3.267	− 0.149	**0.67**	0.105	− 0.056	0.698
My HIV limits me in many things that are important to me	2.3	0.917	1.059	0.433	0.034	**0.673**	0.133	− 0.155	0.615
Because of my HIV, I have grown as a person	3.89	0.854	− 1.001	0.719	**0.712**	0.058	− 0.018	0.064	0.654
Because of my HIV, I know what I want out of life	4.04	0.82	− 1.209	1.621	**0.883**	0.058	− 0.065	− 0.126	0.757
Because of my HIV, I have become a stronger person	4.12	0.716	− 1.123	2.397	**0.802**	− 0.021	− 0.03	0.01	0.731
Because of my HIV, I realise what is really important in life	4.23	0.637	− 1.575	7.562	**0.569**	0.067	0.203	0.263	0.661
Because of my HIV, I have learned a lot about myself	4.27	0.536	0.135	− 0.389	**0.667**	0.061	0.036	0.074	0.658
Because of my HIV, I have learned to work through problems and not just give up	4.24	0.481	0.573	− 0.228	**0.747**	− 0.095	0.035	0.034	0.738
Because of my HIV, I have learned to enjoy the moment more	4.22	0.576	− 0.762	3.469	**0.518**	− 0.047	0.045	0.25	0.653

#### Reiterated confirmatory factor analysis

**Fig. 1:**
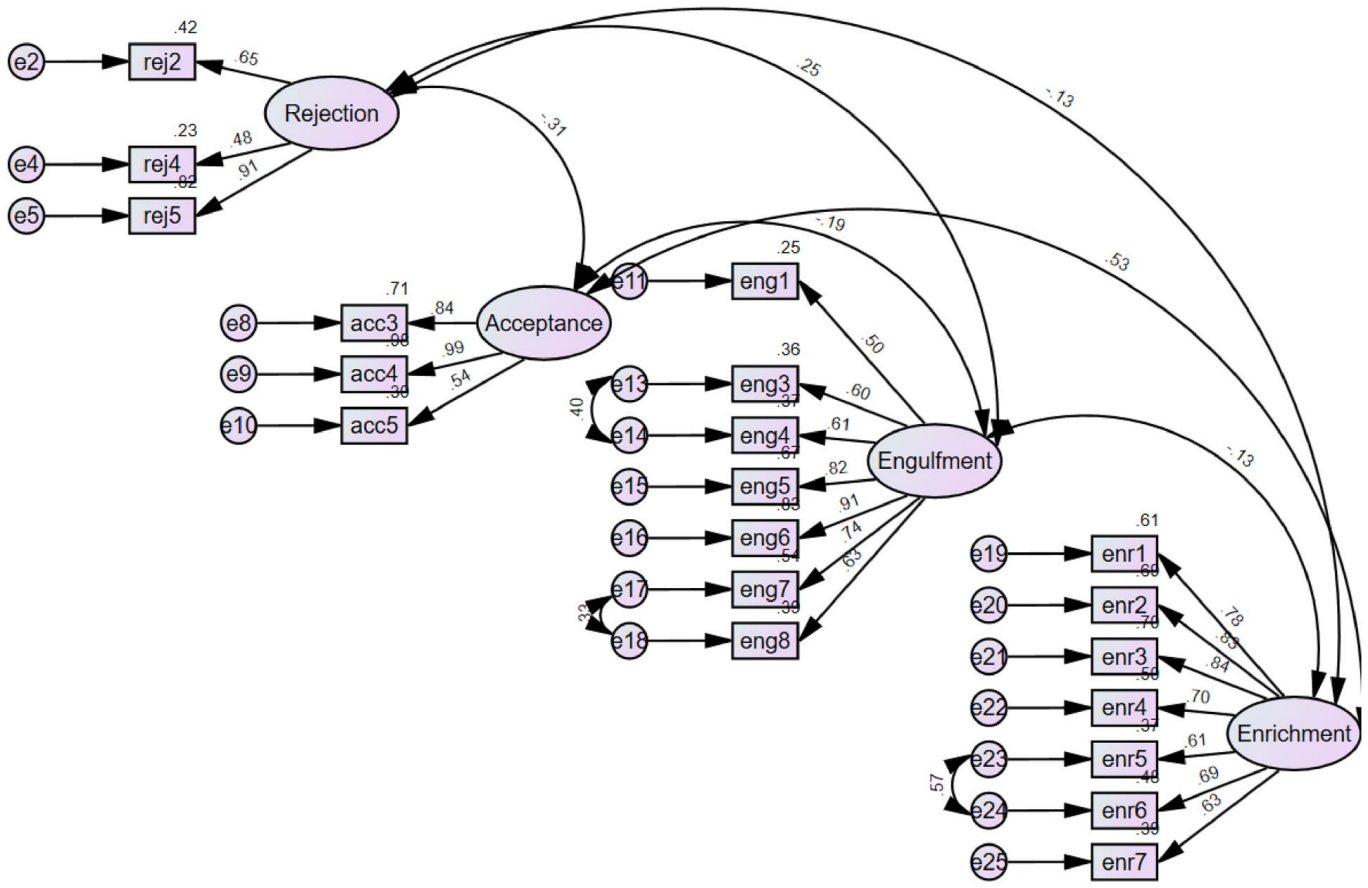
Confirmatory factor analysis: standardised loadings and correlations (e = error terms)

Repeated CFA with the 22-item (model 1) revealed an unacceptable fit (Table 2). A systematic process of deletion of items with poor loadings followed. An initial review of standardised factor loadings of items in model 1 showed that 3 out of 22 items had loadings were below 0.5; *My HIV is part of who I am* (0.44), *My HV has a strong impact on how I see myself* (0.48) and *I hate being talked to about my HIV* (0.48). All the other items had loadings above 0.5. We elected to remove only two of these three items with poor loadings from the model; *My HIV is part of who I am* and *My HV has a strong impact on how I see myself*. The item *I hate being talked to about my HIV,* a rejection item, was retained to maintain the simple structure principle that a factor has to have a minimum of three theoretically meaningful and salient pattern coefficients > 0.4 [66, 71]. The re-specified four-factor model with the remaining 20 items of the IIQ (final model) provided an acceptable fit (Tables 2, 4), this after undertaking an exploratory phase of inspecting the modification indices of all the pairs of error terms and correlating three pairs with the largest indices until the model fitted [2]. This final model had chi-square test statistic = 244.0611, degrees of freedom = 166, probability level ≤ 0.001, normed chi-square = 1.516 and comparative fit index (CFI) = 0.909, RMSEA = 0.076 and SRMR = 0.0893 (Tables 2, 3). The final model included a three-item *acceptance s*ubscale, a three-item *rejection* subscale, a seven-item *enrichment* subscale and a seven-item *engulfment* subscale (Fig. 1). Final factor loadings are presented in Table 4.

#### Correlations, reliability, convergent and discriminant validity

The estimated factor correlations between the four illness identity subscales in Model 2 (20-item) were modest (Table 5) and were all in the expected directions. There was a significant positive correlation between acceptance and enrichment (0.53), a significant negative correlation between acceptance and rejection (− 0.31) and there was also a significant and positive correlation between rejection and engulfment (0.25). There was a non-significant negative correlation between rejection and enrichment (− 0.13), acceptance and engulfment (− 0.19) and enrichment and engulfment were also negatively correlated (− 0.13). Convergent validity was adequate with AVE values for rejection and engulfment, just at the preferred minimum cut-off point of 0.50 (0.49), and for acceptance and enrichment, 0.66 and 0.53 respectively. In addition, composite reliability (CR) values for all subscales were all above 0.60 (Table 5). We also calculated the Cronbach’s alphas of the final four illness identity subscales, they were all 0.7 or above and considered acceptable. Discriminant validity demonstrates that the four illness identity subscales are distinct, largely correlated and have convergent and discriminant validity.

**Table 5:** Convergent and discriminant validity of the final 20-item model

N	Subscale	Number of items	M	SD	Alpha (α)	CR	AVE	MSV	MaxR(H)	1	2	3	4
1	Rejection	3	9.14	2.56	0.7	0.73	0.49	0.1	0.85	*0.7*			
2	Acceptance	3	12.49	1.55	0.83	0.85	0.66	0.28	0.98	− 0.31*	*0.81*		
3	Enrichment	7	29.02	3.63	0.89	0.89	0.53	0.28	0.9	− **0.13**	0.53**	*0.73*	
4	Engulfment	7	15.38	4.72	0.88	0.87	0.49	0.06	0.91	**0.25**^†^	− **0.19**	− **0.13**	*0.7*

Numbers in italics on the diagonal are the square root of the AVE values (discriminant values) and numbers in bold are the correlations among the latent factors

*M* mean, *SD* standard deviation, *CR* composite reliability, *AVE* average variance extracted, *MSV* maximum shared variance

Significance of correlations: ^†^p < 0.100, *p < 0.050, **p < 0.010, ***p < 0.001

#### External validity

##### Association with demographic and illness variables; gender, age and illness duration

We conducted a MANOVA for gender and four illness identity subscales. There was not a significant difference between males and females when considered jointly on acceptance, rejection, engulfment and enrichment, Wilk’s Λ = 0.99, F (4, 85) = 0.25, *P* = 0.91, Partial η^2^ = 0.01. For age and illness duration, a sequence of Spearman rank-order correlations were run in order to determine if there were any relationships between the age of participants and the four illness identity subscales. A two-tailed test of significance indicated that there was no significant relationship between the age of participant and any of the four subscales (Table 6). Correlation analyses were further conducted to ascertain the relationship between duration of illness and the illness identity subscales. With the exception of the acceptance subscale showing a weak positive and significant association with illness duration, r (86) = 0.23, *P* = 0.03, there was no significant association between illness duration and the other three illness identity subscales (Table 6). This result suggest that acceptance of a positive HIV diagnosis is achieved the longer the patient has lived with HIV.

**Table 6:** Correlations: age and illness duration with four illness identity variables; rejection, acceptance, engulfment and enrichment

N	Variable	1	2	3	4	5	6
1	**Age**						
2	**Illness duration**	0.18					
		0.09					
		90					
3	Rejection	− 0.13	− 0.1				
		0.23	0.33				
		90	90				
4	Acceptance	0.00	0.23*	− 0.26*			
		0.96	0.03	0.01			
		90	90	90			
5	Engulfment	− 0.06	− 0.08	0.26*	− 0.32**		
		0.6	0.45	0.01	< 0.001		
		90	90	90	90		
6	Enrichment	− 0.01	0.11	− 0.08	0.44**	− 0.18	
		0.9	0.29	0.46	< 0.001	0.09	
		90	90	90	90	90	

*Correlation is significant at the 0.05 level (2-tailed)

**Correlation is significant at the 0.01 level (2-tailed)

##### External validity: association with disclosure and stigma

To assess the association between disclosure and illness identity a point biserial correlation for disclosure status and the four illness identity subscales was conducted. Results show that rejection and engulfment were negatively correlated and acceptance and enrichment were positively associated with disclosure status. The correlations were however not significant with three of the four illness identity subscales (Table 7). The acceptance subscale was correlated with disclosure status, with a moderate, positive and highly significant association, r (88) = 0.30, p < 0.01 (Table 7). This result suggests that individuals living with HIV who have accepted their illness are likely to have disclosed their positive HIV status to someone else.

**Table 7:** Correlations: disclosure status with four illness identity variables; rejection, acceptance, engulfment and enrichment

N	Variable	1	2	3	4	5
1	Disclosure status					
2	Rejection	− 0.08				
		0.44				
		90				
3	Acceptance	0.30**	− 0.24*			
		< 0.01	0.03			
		90	90			
4	Engulfment	− 0.04	0.31**	− 0.19		
		0.69	< 0.01	0.07		
		90	90	90		
5	Enrichment	0.07	− 0.08	0.49**	− 0.07	
		0.52	0.46	< 0.01	0.5	
		90	90	90	90	

*Correlation is significant at the 0.05 level (2-tailed)

**Correlation is significant at the 0.01 level (2-tailed)

Further correlational analyses were performed to examine the relationships between the four illness identity subscales with participants’ inclination to disclose their HIV status and situational disclosure. We had expected the positive composite of the illness identity measurement (enrichment and acceptance) to be positively associated and the negative illness identity composite (engulfment and rejection) to have a negative relationship with participants’ inclination towards disclosing their HIV status. For the variable *I CAN reveal my HIV status and talk about my HIV to anyone who cares to listen* (reverse coded)*,* the expected result was obtained although the relationships were not significant: rejection r (88) = 0.06, *P* = 0.5, acceptance r (88) = − 0.04, *P* = 0.66, engulfment r (88) = 0.06, *P* = 0.47, and enrichment r (88) = − 0.03, *P* = 0.76 (Table 8). The variable *I can NEVER reveal my HIV status nor talk about my HIV with anyone,* was slightly different and correlated with the rejection subscale, showing a weak positive but highly significant association, r (88) = 0.24, *P* = 0.01 (Table 8). Associations with the other subscales were not significant: acceptance r (88) = − 0.03, *P* = 0.78, engulfment r (88) = 0.06, *P* = 0.46, and enrichment r (88) = − 0.04, *P* = 0.67. This outcome suggests that individuals living with HIV who are rejecting their illness are not likely to talk about or disclose their positive HIV status to someone else. Situational disclosure on the other hand, represented by *I am not afraid to reveal my HIV status or talk about my HIV BUT do so only when it is necessary*, was theorized to be positively associated with enrichment and acceptance and negatively associated with rejection and engulfment. There were no significant relationships of this variable with any of the illness identity subscales (Table 8).

**Table 8:** Correlations: inclination to disclose positive HIV status and situational disclosure with four illness identity subscales; rejection, acceptance, engulfment and enrichment

N	Variable	1	2	3	4	5	6	7
	Illness identity							
1	Rejection							
2	Acceptance	− 0.22*						
		0.01						
		90						
3	Engulfment	0.20*	− 0.28**					
		0.01	< 0.001					
		90	90					
4	Enrichment	− 0.06	0.38^**^	− 0.14				
		0.47	< 0.001	.08				
		90	90	90				
	Disclosure							
5	HIV status reveal to anyone	0.06	− 0.04	0.06	− 0.03			
		0.5	0.66	0.47	0.76			
		90	90	90	90			
6	HIV status never reveal	− 0.24**	0.03	0.06	− 0.04	− 0.16		
		0.01	0.78	0.46	0.67	0.08		
		90	90	90	90	90		
7	HIV status reveal when necessary	0.14	− 0.11	0.14	0.03	0.23*	− 0.04	
		0.11	0.23	0.11	0.71	0.01	0.66	
		90	90	90	90	90	90	

*Correlation is significant at the 0.05 level (2-tailed)

**Correlation is significant at the 0.01 level (2-tailed)

Additionally, a series of Spearman rank-order correlations were further performed in order to determine the relationship among the four HIV stigma subscales and the four illness identity subscales. Two-tailed tests of significance indicated that all but three relationships among the stigma and illness identity subscales were significant (Table 9). These results suggest that patients living with HIV who reject and are engulfed by their illness are likely to have a negative self-image, express concerns about disclosing their positive HIV status, and be concerned about public attitudes towards PLHIV. In addition, those who reject their illness are likely to report personalised stigma. On the other hand, those who have accepted and have reached an enrichment illness state, are not likely to have a negative self-image nor report personalised stigma. In addition, those who have accepted their illness, are not likely to communicate concerns about disclosing their positive HIV status.

**Table 9:** Correlation: HIV stigma with four illness identity variables; rejection, acceptance, engulfment and enrichment

N	Variable	1	2	3	4	5	6	7	8
	Illness identity								
1	Rejection								
2	Acceptance	− 0.26*							
		0.01							
		90							
3	Engulfment	0.26*	− 0.32^**^						
		0.01	< 0.001						
		90	90						
4	Enrichment	− 0.08	0.44**	− 0.18					
		0.46	< 0.001	0.09					
		90	90	90					
	HIV stigma								
5	Personalized stigma	0.18	− 0.39**	0.38**	− 0.36**				
		0.1	< 0.001	< 0.001	< 0.001				
		85	85	85	85				
6	Disclosure concerns	0.34**	− 0.43**	0.37**	− 0.16	0.27*			
		< 0.001	< 0.001	< 0.001	0.14	0.01			
		90	90	90	90	85			
7	Concerns about public attitudes	0.26*	− 0.18	0.32**	− 0.09	0.23*	0.61**		
		0.01	0.08	< 0.001	0.41	0.03	< 0.001		
		90	90	90	90	85	90		
8	Negative self-image	0.27*	− 0.30**	0.55**	− 0.30**	0.35**	0.47**	0.59**	
		0.01	< 0.001	< 0.001	< 0.001	< 0.001	< 0.001	< 0.001	
		90	90	90	90	85	90	90	

## Discussion

We highlighted the paucity of information on and the importance of measurement of illness identity in PLHIV in high prevalence and resource constrained settings such as South Africa. In response, we first sought to adapt the IIQ to this context to adjust for the expected differences in definitions, beliefs and behaviour related to the constructs within the IIQ between the Belgian and South African contexts. Subsequently, the factorial and overall validity of the original 25-item IIQ [39, 46, 47] was conducted using a sample of South African PLHIV. This is the first study that demonstrates the concept of illness identity, using a substantially stigmatised chronic illness, HIV, and contextualised within a high prevalence LMIC context.

### Omitted items

Of the 25 original IIQ items, five were eventually excluded from the adapted IIQ due to either semantic insufficiency or inadequate measurement equivalence due to loss of meaning and/or poor and complex factor loadings. Specifically, one excluded item, *I never talk to others about my HIV*, a rejection item, may have been an inappropriate statement to determine the level of rejection in this cohort of participants. This is because respondents for this validation study were recruited from primary healthcare facilities during their routine consultation for HIV care and management. They were at the facility to “talk about their HIV”. As such, it would have been counterintuitive for the participants to especially respond to this statement in the affirmative. For the current context, the statement required being qualified with a preamble “with the exception of my healthcare providers, I never to talk to others about my HIV”.

It is also plausible that the meaning of the next eliminated engulfment item *My HIV has a strong impact on how I see myself* may have been interpreted in an HIV identity centrality sense. HIV identity centrality is a somewhat similar but unrelated concept, defined as the degree to which PLHIV consider being HIV-positive a part of who they are as a person [51]. It has been shown to act as a buffer against the association between anticipated stigma and HIV symptoms for PLHIV [14]. Indeed, one of the items within the four-item HIV identity centrality sub-scale reads *In general, my HIV-status is an important part of the way I see myself*. The two items are from two different identity subscales but are very similar in both wording and meaning. We believe this may have been a case of misinterpretation because by description, individuals who are engulfed by their chronic illnesses completely define themselves in terms of their illness, although with negative consequences [47]. Therefore, engulfment and identity centrality may be two overlapping concepts that, depending on the context of the illness, one may be misconstrued for the other.

The final three deleted items which included two acceptance items; *my HIV simply belongs to me as a person*, *my HIV is part of who I am,* and a rejection item: *I refuse to see my HIV as part of myself*, all have a connotation of “ownership” of the illness. Along with ownership, within the meaning of the three items there is a potential objectification of HIV into a possession or belonging. Acquiring chronic illness, particularly a type that is highly stigmatised such as HIV, then becomes synonymous with acquisition of an undesired possession [68]. The question then becomes how do PLHIV acknowledge or accept the unwanted possession as their own. The difficulty of accepting and fully taking ownership of and integrating into the self a particularly stigmatised identity, such as that of HIV, is aptly captured by Wouters & De Wet [74] using Goffman’s [21] conceptualization of a ‘moral career’. Researchers argue that for PLHIV, the path towards assumption of ownership of this unwelcome belonging or integration of HIV into identity is complex, contextual and not as straightforward and requires engagement with the illness [68, 74]. That as PLHIV may attempt and manage to integrate the illness into their identity, there may exist a consistent conflict within themselves, mainly driven by stigma and other structural barriers. This results in patients vacillating between incorporation and non-incorporation accompanied by identity evaluation, adjustments and improvements, and therefore becomes the ‘moral career’ of PLHIV [74]. This provocation of ownership that PLHIV are confronted with, potentially experienced by the participants in this study, challenges the applicability of the three ‘ownership’ items within the current study context. Accordingly, their problematic loadings may potentially be explained by this phenomenon.

In essence, exclusion of the five items as described above from the adapted IIQ underscores the importance of contextualizing scale items in context and the necessity for adequate adaptations of measurement instruments, particularly when used across different languages, time and settings than those for which they were originally developed and tested.

### External validity

Consistent with previous literature, gender [39, 46] and age [47] did not influence PLHIV’s illness integration into identity. While some evidence suggests that the incorporation of a chronic illness into identity is a recursive, non-linear and continuous process [48, 63, 68, 74] and that time since diagnosis was irrelevant to the construction of an illness identity [4], we found that acceptance was associated with illness duration. This finding is consistent with the original validation study [46] and other studies that have demonstrated how over time, although timing is not well-defined, nor is the process linear, there is acceptance of illness and eventually people manage to integrate HIV into their identity [7, 8, 29].

We also assessed the practical and clinical applicability of the concept of illness identity in PLHIV by correlating the four illness identity subscales with aspects of disclosure and HIV related stigma. Rejection and engulfment were particularly salient in association with overall HIV related stigma and aversion towards disclosing a positive HIV status. Acceptance and enrichment were negatively associated with all aspects of HIV related stigma. In addition, acceptance was associated with having disclosed to someone. These findings are consistent with research which demonstrates that acceptance of an HIV diagnosis, HIV related stigma and disclosure of positive HIV status may buffer against HIV related stigma and as a result facilitate disclosure of HIV status [29, 45, 74]. Baumgartner [7] also argued that the type of disclosure, partial, public or as guided by context, may be used as a proxy for the level of integration of HIV into identity.

## Limitations

At a point during the initial period of baseline data collection activities described in the methods had to be suspended due to the global COVID-19 pandemic. As a result, a passable sample, sufficient to conduct the validation study with our Afrikaans speaking respondents included, could not be obtained. In addition, we achieved a smaller than intended sample size for our isiXhosa speaking participants. A larger and more inclusive sample could have rendered the results of this validation richer and more meaningful. Despite this we have demonstrated that for our purposes and objectives, the sample size used was adequate.

Because disclosure of HIV status is vital to the HIV illness trajectory, statements that specifically solicited information on talking about HIV with others need to be qualified by mentioning categories of individuals that participants were willing to talk to about their HIV, particularly as it pertains to health professionals and or other caregivers.

Participants for the current study were recruited from health facilities while actually seeking care for their HIV and therefore it is hard to assume they were rejecting their HIV and were possibly accepting their diagnosis. This may have introduced some sampling bias because only people in care were included and may have confounded the results. Future research exploring illness identity in PLHIV should expand the sampling frame beyond care settings to obtain a more varied sample. In addition, we tested the scale in a sample of relatively recently diagnosed HIV positive adults who sought care. Because of this selection procedure, certain elements of incorporating HIV into one’s identity will thus be similar for all these individuals. Future studies should thus repeat the analyses for other HIV-positive populations such as experienced patients.

## Conclusion

Illness identity is a fundamental concept useful to understanding the chronic illness trajectory and its outcomes. The development of a context specific and appropriate tool to measure illness identity in PLHIV is a significant stride because it enables an understanding of why some live well with HIV and why others do not. For those involved in the care and management of PLHIV, measuring illness identity further provides an alternative option for the exploration of how outcomes for PLHIV such as adherence to treatments, could be improved and sustained.

A significant aspect of this study was to adapt a chronic illness identity measurement instrument, the IIQ, to a new context and type of chronic illness. Besides the task of contextualising the instrument, the validity of the construct in the new context had to be demonstrated. We have presented evidence of a four-factor structure with good reliability of the four subscales of illness identity; rejection, acceptance, engulfment and enrichment*,* using a sample of South African PLHIV. Fundamentally, the successful cross-cultural validation and subsequent availability of a validated instrument capable of measuring illness identity, not only addresses the paucity of information on the subject, but also expands on our knowledge about illness identity, specifically as it relates to HIV. At a practical level, the cross-cultural adaptation and validation of the IIQ has yielded a validated instrument that measures illness identity in IsiXhosa speaking adults living with HIV in South Africa. This will mitigate the risk of introducing bias into the tool with future use.

## Data Availability

The data that support the findings are part of the ongoing Sinako cluster-randomised controlled trial (Pan African Clinical Trial Registry, PACTR201906476052236), a study on HIV competent households in South Africa [41, 42] and as such are under embargo. The said data will however be available on request from the authors upon completion of the trial.

## Appendix

See Table 10.

**Table 10:** Process model of cross-cultural adaptation of an instrument, adapted from Herdman et al. [26] and Reichenheim and Moraes [52]

Aspect	Definition	Evaluation strategy	Possible outcomes of the evaluation
Conceptual equivalence	When the domains that represent the construct under study (e.g. engulfment, rejection, acceptance and enrichment domains within the IIQ) not only have the same relationship in both the original and target context but the weight placed on each domain is also the same	Review of literature on the concept under study and or its domain, published in both the original and target context	Domains are relevant to the target context and weight placed on each is the same, thus the construct may be deemed valid in the target context
	Through conceptual equivalence, researchers can determine whether adapting the questionnaire is warranted or not	Exploration of how the domains under study are interpreted by the target population	Domains are relevant to the target context. However, weight placed on each is different
		Discussion with experts and specialists in the field	Some of the domains are irrelevant in the target context, therefore only limited adaptation will be possible
			All domains are irrelevant and as such adaptation cannot be justified
Item equivalence	When items that measure a latent trait (e.g. *acceptance* as one of the domains of illness identity in the current study) mean the same thing in both the original and the target context. Relevance and acceptability of items must also be the same in both target and original contexts	Review of available relevant literature	Item amendment not required and can be used as is in the target context
		Exploration of how the items that measure the latent trait are interpreted by the target population	Although minor item adjustment required, items can still be used to a large degree in target context as was in the original context
		Psychometric testing (e.g. Rasch item analysis)	There is a need to substitute some of the items
			Both original and replaced items do not measure the latent trait, are unacceptable and irrelevant
Semantic equivalence	The same *meaning* of items in the original language (English in the current study) could be established in the target languages (IsiXhosa and Afrikaans) and in so doing, simultaneously attaining the same effect on respondents in the target context	First determining meaning of key words or phrases used within the instrument in the source language	Items are easy, difficult or impossible to translate
		Actual translation and where necessary, adjusting the level of language to that of the target population	
		Translator being aware of the target population and as such adjusting the language of the instrument to the dialect of the target population	
Operational equivalence	When mode of administration, format, instructions and measurement of the instrument can be applied in the same manner in the target context as was in the original context	Assessment of literacy levels of the target population will guide and inform operationalization of the instrument	Mode of administration, format, instructions and measurement of the instrument can be applied in the same way in the target population as was in the original context
		Review of cultural norms of the target population	Only after adjusting some aspects of operationalisation (e.g. mode of administration) can the instrument be used in the target context
		Actual testing of suggested methods within a sample of target population	Operational equivalence cannot be achieved
Measurement Equivalence	Assesses whether the psychometric properties (reliability, responsiveness and construct validity) of the translated version of the instrument are at an appropriate level	Cronbach’s α	Properties are the same, different or systematically different
		Intra-class correlation coefficient	
		Paired t-statistic	
		Effect size statistic	
		Responsiveness statistic	
		Factor analysis	

## References

[1] Adams S, Pill R, Jones A, Adams S, Pill O, Jones A (1997). Medication, chronic illness and identity: the perspective of people with asthma. Soc Sci Med.

[2] Alavi M, Visentin DC, Thapa DK, Hunt GE, Watson R, Cleary M (2020). Chi-square for model fit in confirmatory factor analysis. J Adv Nurs.

[3] Audet CM, Wagner LJ, Wallston KA (2015). Finding meaning in life while living with HIV: validation of a novel HIV meaningfulness scale among HIV-infected participants living in Tennessee. BMC Psychol.

[4] Aujoulat I, Luminet O, Deccache A (2007). The perspective of patients on their experience of powerlessness. Qual Health Res.

[5] Aujoulat I, Marcolongo R, Bonadiman L, Deccache A (2008). Reconsidering patient empowerment in chronic illness: a critique of models of self-efficacy and bodily control. Soc Sci Med.

[6] Bartlett M (1954). A note on the multiplying factors for various χ^2^ approximations. J R Stat Soc Ser B Methodol.

[7] Baumgartner LM (2007). The incorporation of the HIV/AIDS identity into the self over time. Qual Health Res.

[8] Baumgartner LM (2012). The perceived effect of time on HIV/AIDS identity incorporation. Qual Rep.

[9] Berger B, Ferrans C, Lashley F (2001). Measuring stigma in people with HIV: psychometric assessment of the HIV stigma scale. Res Nurs Health.

[10] Charmaz K (1997). Good days, bad days.

[11] Deeks SG, Lewin SR, Havlir DV (2013). The end of AIDS: HIV infection as a chronic disease. Lancet.

[12] Degroote S, Vogelaers D, Vandijck DM (2014). What determines health-related quality of life among people living with HIV: an updated review of the literature. Arch Public Health.

[13] du Plessis G (2011). Individual responsibility for health and HIV Infection: a critical investigation of the lived experience of HIV-positive women. S Afr J Psychol.

[14] Earnshaw VA, Lang SM, Lippitt M, Jin H, Chaudoir SR (2015). HIV stigma and physical health symptoms: do social support, adaptive coping, and/or identity centrality act as resilience resources?. AIDS Behav.

[15] Engel GL (1977). The need for a new medical model: a challenge for biomedicine. Science.

[16] Fetvadjiev VH, Van De Vijver FJR (2016). Measures of personality across cultures measures of cross-cultural values, personality and beliefs. Measures of personality and social psychological constructs.

[17] Finkelstein-Fox L, Park CL, Kalichman SC (2020). Health benefits of positive reappraisal coping among people living with HIV/AIDS: a systematic review. Health Psychol Rev.

[18] Flowers P, Davis M, Hart G, Rosengarten M, Frankis J, Imrie J (2006). Diagnosis and stigma and identity amongst HIV positive Black Africans living in the UK. Psychol Health.

[19] Friedland BA, Gottert A, Hows J, Baral SD, Sprague L, Nyblade L (2020). The people living with HIV stigma index 2.0: generating critical evidence for change worldwide. AIDS.

[20] Gjersing L, Caplehorn JR, Clausen T (2010). Cross-cultural adaptation of research instruments: language, setting, time and statistical considerations. BMC Med Res Methodol.

[21] Goffman E (1959). The moral career of the mental patient. Psychiatry.

[22] Golub SA, Gamarel KE, Rendina HJ (2014). Loss and growth: identity processes with distinct and complementary impacts on well-being among those living with chronic illness. Psychol Health Med.

[23] Goudge J, Ngoma B, Manderson L, Schneider H (2009). Stigma, identity and resistance among people living with HIV in South Africa. SAHARA-J J Soc Asp HIV/AIDS Res Alliance.

[24] Graffigna G, Barello S, Riva G, Bosio AC (2014). Patient engagement: the key to redesign the exchange between the demand and supply for healthcare in the era of active ageing. Stud Health Technol Inform.

[25] Hatala AR, Bird-Naytowhow K, Pearl T, Peterson J, del Canto S, Rooke E, Calvez S, Meili R, Schwandt M, Mercredi J, Tait P (2018). Being and becoming a helper: illness disclosure and identity transformations among indigenous people living with HIV or AIDS in Saskatoon, Saskatchewan. Qual Health Res.

[26] Herdman M, Fox-Rushby J, Badia X (1998). A model of equivalence in the cultural adaptation of HRQoL instruments: the universalist approach. Qual Life Res.

[27] Hernandez CANN (1995). The experience of living with insulin-dependent diabetes: lessons for the diabetes educator. Diabetes Educ.

[28] Ho LPP, Goh ECL (2020). “I have HIV but I’m not the HIV”—the experiences of heterosexual Chinese men living with HIV in Singapore. AIDS Care.

[29] Horter S, Thabede Z, Dlamini V, Bernays S, Stringer B, Mazibuko S, Dube L, Rusch B, Jobanputra K (2017). “Life is so easy on ART, once you accept it”: acceptance, denial and linkage to HIV care in Shiselweni, Swaziland. Soc Sci Med.

[30] Hu LT, Bentler PM (1999). Cutoff criteria for fit indexes in covariance structure analysis: conventional criteria versus new alternatives. Struct Equ Model.

[31] Hui CH, Triandis HC (1985). Measurement in cross-cultural psychology: a review and comparison of strategies. J Cross Cult Psychol.

[32] Izquierdo I, Olea J, Abad FJ (2014). El análisis factorial exploratorio en estudios de validación: Usos y recomendaciones. Psicothema.

[33] Jacobs GB, de Beer C, Fincham JE, Adams V, Dhansay MA, van Rensburg EJ, Engelbrecht S (2006). Serotyping and genotyping of HIV-1 infection in residents of Khayelitsha, Cape Town, South Africa. J Med Virol.

[34] Kaiser HF (1974). An index of factorial simplicity. Psychometrika.

[35] Kralik D (2002). The quest for ordinariness transition experienced by midlife women living with chronic illness. J Adv Nurs.

[36] Kralik D, Koch T, Price K, Howard N (2004). Chronic conditions and self management: taking action to create order. J Clin Nurs.

[37] Laws MB (2016). Explanatory models and illness experience of people living with HIV. AIDS Behav.

[38] Leventhal H, Halm E, Horowitz C, Leventhal E, Ozakinci G (2004). Living with chronic illness: a contextualised, self-regulation approach. The Sage handbook of health psychology.

[39] Luyckx K, Oris L, Raymaekers K, Rassart J, Moons P, Verdyck L, Mijnster T, Mark RE (2018). Illness identity in young adults with refractory epilepsy. Epilepsy Behav.

[40] Mahungu TW, Rodger AJ, Johnson MA (2009). HIV as a chronic disease. Clin Med.

[41] Masquillier C, Knight L, Campbell L, Sematlane N, Delport A, Dube T, Wouters E (2020). Sinako, a study on HIV competent households in South Africa: a cluster-randomised controlled trial protocol. Trials.

[42] Masquillier C, Wouters E, Campbell L, Delport A, Sematlane N, Dube T, Knight L (2020). Households in HIV care: designing an intervention to stimulate HIV competency in households in South Africa. Front Public Health.

[43] Molina L, Albir A (2002). Translation techniques revisited. A dynamic and functionalist approach. Meta Transl J.

[44] Morea JM, Friend R, Bennett RM (2008). Conceptualizing and measuring illness self-concept: a comparison with self-esteem and optimism in predicting fibromyalgia adjustment. Res Nurs Health.

[45] Nam SL, Fielding K, Avalos A, Dickinson D, Gaolathe T, Geissler PW (2008). The relationship of acceptance or denial of HIV-status to antiretroviral adherence among adult HIV patients in urban Botswana. Soc Sci Med.

[46] Oris L, Luyckx K, Rassart J, Goubert L, Goossens E, Apers S, Arat S, Vandenberghe J, Westhovens R, Moons P (2018). Illness identity in adults with a chronic illness. J Clin Psychol Med Settings.

[47] Oris L, Rassart J, Prikken S, Verschueren M, Goubert L, Moons P, Berg CA, Weets I, Luyckx K (2016). Illness identity in adolescents and emerging adults with type 1 diabetes: introducing the illness identity questionnaire. Diabetes Care.

[48] Paterson (2001). The shifting perspectives model of chronic illness. J Nurs Scholarsh.

[49] Paterson DL, Swindells S, Mohr J, Brester M, Vergis EN, Squier C, Wagener MM, Singh N (2013). Adherence to anti-HIV therapy and the outcome of treatment. Ann Intern Med.

[50] Perazzo J, Reyes D, Webel A (2017). A systematic review of health literacy interventions for people living with HIV. AIDS Behav.

[51] Quinn DM, Earnshaw VA (2011). Understanding concealable stigmatized identities: the role of identity in psychological, physical, and behavioral outcomes. Soc Issues Policy Rev.

[52] Reichenheim ME, Moraes CL (2007). Operationalizing the cross-cultural adaptation of epidemological. Rev Saude Publica.

[53] Reinius M, Wettergren L, Wiklander M, Svedhem V, Ekström AM, Eriksson LE (2017). Development of a 12-item short version of the HIV stigma scale. Health Qual Life Outcomes.

[54] Rohleder P, Gibson K (2006). We are not fresh’: HIV-positive women talk of their experience of living with their ‘spoiled identity. S Afr J Psychol.

[55] Rushby L. LibGuides: social development: government information: library guide: community profile statistics. 2019. http://libguides.lib.uct.ac.za/c.php?g=214526&p=1433767.

[56] Simoni JM, Amico KR, Pearson CR, Malow R (2008). Strategies for promoting adherence to antiretroviral therapy: a review of the literature. Curr Infect Dis Rep.

[57] Soskolne T. Moving beyond the margins: a narrative analysis of the life stories of women living with HIV/AIDS in Khayelitsha. University of Cape Town; 2003. http://hdl.handle.net/11427/19750.

[58] Soskolne T, Stein J, Gibson K. Working with ambivalence: finding a positive identity for HIV/AIDS in South Africa. University of Cape Town; 2003. http://hdl.handle.net/11427/19726.

[59] Sperber AD, Devellis RF, Boehlecke B (1994). Cross-cultural translation: methodology and validation. J Cross Cult Psychol.

[60] Sprangers MAG, Schwartz CE (1999). Integrating response shift into health-related quality of life research: a theoretical model. Soc Sci Med.

[61] Stinson K, Goemaere E, Coetzee D, Van Cutsem G, Hilderbrand K, Osler M (2017). Cohort profile: the Khayelitsha antiretroviral programme, Cape Town, South Africa. Int J Epidemiol.

[62] Tait EF. An investigation into the lived experiences of HIV-positive African Women, (September). Doctoral dissertation, University of East London; 2013.

[63] Telford K, Kralik D, Koch T (2006). Acceptance and denial: implications for people adapting to chronic illness: literature review. J Adv Nurs.

[64] Tewksbury R, McGaughey D (1998). Identities and identity transformations among persons with HIV disease. Int J Sex Gend Stud.

[65] Thompson MA, Mugavero MJ, Amico KR, Cargill VA, Chang LW, Gross R, et al. Guidelines for improving entry into and retention in care and antiretroviral adherence for persons with HIV: evidence-based recommendations from an International Association of Physicians in AIDS Care panel. Ann Internal Med. 2012;156(11):817–833, W-284, W-285, W-286, W-287, W-288, W-289. 10.7326/0003-4819-156-11-201206050-00419. 10.7326/0003-4819-156-11-201206050-00419 PMC4044043 22393036

[66] Thurstone LL (1931). Multiple factor analysis. Psychol Rev.

[67] Tilden B (2005). Identity and adherence in a diabetes patient: transformations in psychotherapy. Qual Health Res.

[68] Tsarenko Y, Polonsky MJ (2011). “You can spend your life dying or you can spend your life living”: identity transition in people who are HIV-positive. Psychol Health.

[69] Van Bulck L, Goossens E, Luyckx K, Oris L, Apers S, Moons P (2018). Illness identity: a novel predictor for healthcare use in adults with congenital heart disease. J Am Heart Assoc.

[70] Van Bulck L, Luyckx K, Goossens E, Oris L, Moons P (2019). Illness identity: capturing the influence of illness on the person’s sense of self. Eur J Cardiovasc Nurs.

[71] Watkins MW (2018). Exploratory factor analysis: a guide to best practice. J Black Psychol.

[72] Whittemore R, Roy SC (2002). Adapting to diabetes mellitus: a theory synthesis. Nurs Sci Q.

[73] Wouters E (2012). Life with HIV as a chronic illness: a theoretical and methodological framework for antiretroviral treatment studies in resource-limited settings. Soc Theory Health.

[74] Wouters E, De Wet K (2016). Women’s experience of HIV as a chronic illness in South Africa: hard-earned lives, biographical disruption and moral career. Sociol Health Illn.

